# Lack of association between *IL10* polymorphisms and sarcoidosis in Japanese patients

**Published:** 2012-01-25

**Authors:** Kenichi Sakuyama, Akira Meguro, Masao Ota, Mami Ishihara, Riyo Uemoto, Haruyasu Ito, Eiichi Okada, Kenichi Namba, Nobuyoshi Kitaichi, Shin-ichiro Morimoto, Toshikatsu Kaburaki, Yasutaka Ando, Shinobu Takenaka, Takenosuke Yuasa, Shigeaki Ohno, Hidetoshi Inoko, Nobuhisa Mizuki

**Affiliations:** 1 Department of Ophthalmology and Visual Science, Yokohama City University Graduate School of Medicine, Yokohama, Kanagawa, Japan; 2 Department of Legal Medicine, Shinshu University School of Medicine, Matsumoto, Nagano, Japan; 3 Okada Eye Clinic, Yokohama, Kanagawa, Japan; 4 Department of Ophthalmology, Hokkaido University Graduate School of Medicine, Sapporo, Hokkaido, Japan; 5 Department of Ophthalmology, Health Sciences University of Hokkaido, Sapporo, Hokkaido, Japan; 6 Division of Cardiology, Department of Internal Medicine, Fujita Health University School of Medicine, Toyoake, Aichi, Japan; 7 Department of Ophthalmology, University of Tokyo School of Medicine, Bunkyo-ku, Tokyo, Japan; 8 Department of Ophthalmology, Kitasato Institute Hospital, Minato-ku, Tokyo, Japan; 9 Department of Ophthalmology, Keio University School of Medicine, Shinjuku-ku, Tokyo, Japan; 10 Department of Respiratory Diseases, Kumamoto City Hospital, Kumamoto, Kumamoto, Japan; 11 Yuasa Eye Clinic, Osaka, Japan; 12 Department of Ocular Inflammation and Immunology, Hokkaido University Graduate School of Medicine, Sapporo, Hokkaido, Japan; 13 Department of Molecular Life Science, Division of Molecular Medical Science and Molecular Medicine, Tokai University School of Medicine, Isehara, Kanagawa, Japan

## Abstract

**Purpose:**

To investigate whether interleukin 10 (*IL10*) gene polymorphisms are associated with the development of sarcoidosis in Japanese patients.

**Methods:**

Two hundred and eighty-eight Japanese sarcoidosis patients and 310 Japanese healthy controls were recruited. We genotyped 9 single-nucleotide polymorphisms in *IL10* and assessed the allelic diversity between cases and controls.

**Results:**

No significant differences in the frequency of *IL10* alleles, genotypes, and haplotypes in the sarcoidosis cases compared to the controls were detected.

**Conclusions:**

Our results suggest that *IL10* polymorphisms are not significantly related to the pathogenesis of sarcoidosis in the Japanese population.

## Introduction

Sarcoidosis is a systemic inflammatory disorder characterized by non-caseating granuloma formation in many organs, such as: lung, skin, eye, lymph nodes, central and peripheral nervous system, and heart [1-3].

In Japan, the reported incidence rate of the disease is 1.01 per 100,000 inhabitants [4]. On a global scale, this incidence rate is low. African Americans incidence rate of the disease is 35.5 per 100,000. That of Caucasian Americans is 10.9 per 100,000 [5]. Japanese patients have a higher likelihood of ocular involvement compared with other ethnic groups [4,6]. According to a recent epidemiological study of sarcoidosis in Japan, patients with ocular involvement was 54.8% of cases and impaired vision was the most frequent symptom (28.8%) [4]. In European patients, erythema nodosum of skin lesions is commonly seen. It is rare in Japanese patients [7]. This way, the frequency and course of sarcoidosis varies widely among racial groups. It supports the assumption that some predisposing genetic factors play roles in the development of the disease. There is also evidence supporting a possibility of association with genetic factors. Some familial sarcoidosis cases [8], and associations between the disease and human leukocyte antigen (HLA) systems were reported [9,10]. The exact cause of the disease remains undetermined, but it is currently thought that genetic factors may be the basis of disease susceptibility.

It is also thought that environmental factors associate with the disease progression. By using polymerase-chain-reaction (PCR) techniques, *Mycobacterium tuberculosis* and *Propionibacterium acnes* DNA have been detected in sarcoid lesions [11-14]. Recent studies have shown that serum samples from sarcoidosis patients contain antibodies against mycobacterium antigens [15].These studies suggest that immune responses to bacterial infections can affect the development of sarcoidosis.

The inflammatory response in sarcoidosis is characterized by the increased production of several inflammatory cytokines produced by type 1 helper T (Th1) cells and macrophages, such as interleukin-2 (IL-2), interferon-gamma (IFN-γ), and tumor necrosis factor alpha (TNF-α) [16-18]. These cytokines seem to play a roll leading to the formation of granuloma [3]. In some of sarcoidosis patients, the granulomatous response resolves, and the remaining patients have chronic disease include fibrosis. Interleukin-10 (IL-10) produced by type 2 helper T (Th2) cells is associated with the resolution [3]. IL-10 is a potent suppressor of these inflammatory cytokines [19]. Several studies reported that changes in cytokine production might have been caused by a genetic polymorphisms and some of them might be involved in disease susceptibility and progression. Recent studies have reported that *IL10* polymorphisms were associated with several inflammatory diseases [20-22]. In the present study, we evaluated the association of multiple SNPs in *IL10* in Japanese sarcoidosis patients.

## Methods

### Subjects

Two hundred and eighty-eight unrelated patients with a diagnosis of sarcoidosis and 310 healthy controls were recruited from Yokohama City University, Hokkaido University, Fujita Health University, Tokyo University, Keio University, Kumamoto City hospital and Yuasa eye clinic. All patients and control participants were of Japanese ethnicity. Sarcoidosis patients were diagnosed according to the diagnostic criteria developed by the Japanese Society of Sarcoidosis and Other Granulomatous Disorders (JSSOG) previously described [23]. Uveitis with sarcoidosis was assessed based on the “Guidelines for Diagnosis of Ocular Lesions in Sarcoidosis” prepared by the JSSOG. The ocular features of sarcoidosis were defined as granulomatous uveitis plus two or more of the following: infiltration of the anterior chamber (mutton-fat keratic precipitates/iris nodules), trabecular meshwork nodules and/or tent-shaped peripheral anterior synechia, masses of vitreous opacities (snowball-like or string of pearls-like appearance), periphlebitis with perivascular nodules; multiple candle-wax type chorioretinal exudates and nodules, and/or laser photocoagulation spot-like chorioretinal atrophy. All subjects had a similar social background and resided in the same urban area. The research methods were in compliance with the guidelines of the Declaration of Helsinki. Details of the study were explained to all patients and controls, and valid consent for genetic screening was obtained.

### *IL10* genotyping

**Figure 1:**
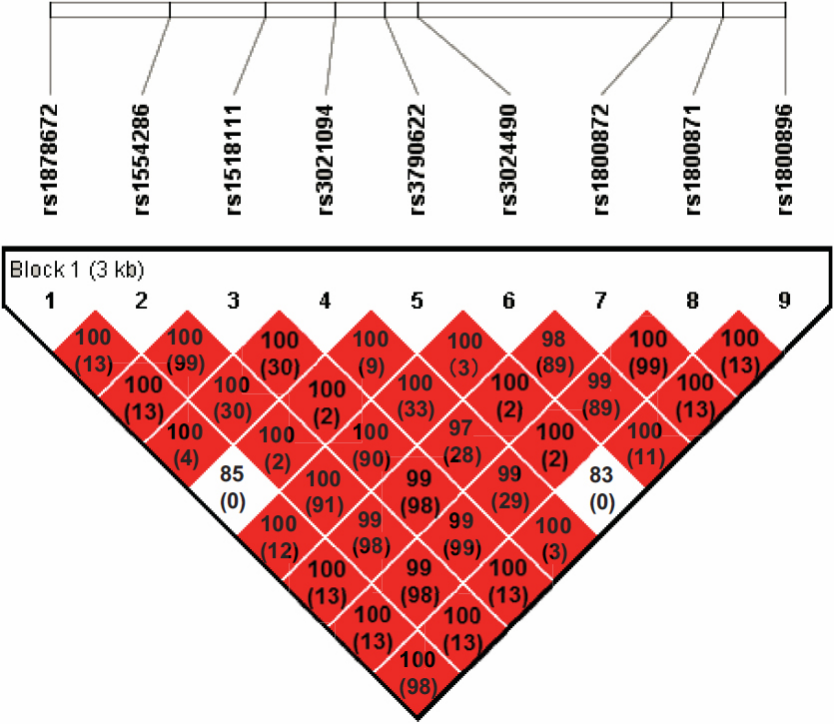
Linkage disequilibrium plot of nine SNPs in *IL10* SNPs in 598 study participants. The D’ value and r^2^ value (in parentheses) corresponding to each SNP pair are expressed as a percentage and shown within the respective square. Higher D’ is indicated by a brighter red. The 9 SNPs constitute a haplotype block spanning 3.2 kb of *IL10*.

Peripheral blood lymphocytes were collected, and genomic DNA was extracted from peripheral blood cells using the QIAamp DNA Blood Maxi Kit (Qiagen, Tokyo, Japan). We selected *IL10* SNPs which previously showed a significant association with Japanese Behcet’s disease [22]: rs1878672, rs1554286, rs1518111, rs3021094, rs3790622, rs3024490, rs1800872, rs1800871, and rs1800896 (Figure 1, Table 1). Genotyping of all SNPs was performed using the TaqMan 5′exonuclease assay using primers supplied by Applied Biosystems (Foster City, CA). Probe fluorescence signals were detected by TaqMan Assay for real-time PCR (7500 Real Time PCR System; Applied Biosystems) following the manufacturer’s instructions.

**Table 1:** Allele frequencies of SNPs of the *IL10* gene among cases and controls.

** **	** **	** **	** **	**Minor allele frequency, n (%)**				** **	** **
**rsID**	**Position Build 37.1**	**Location**	**Allele 1>2**	**Cases n=288**		**Controls n=310**		**p**	**OR (95% CI)**
rs1878672	chr1: 206,943,713	Intron 3	C>G	31	(5.4)	41	(6.7)	0.356	0.80 (0.49–1.29)
rs1554286	chr1: 206,944,233	Intron 3	T>C	181	(31.4)	210	(34.0)	0.347	0.89 (0.70–1.13)
rs1518111	chr1: 206,944,645	Intron 2	A>G	180	(31.3)	210	(34.1)	0.296	0.88 (0.69–1.12)
rs3021094	chr1: 206,944,952	Intron 1	A>C	224	(38.9)	231	(37.5)	0.622	1.06 (0.84–1.34)
rs3790622	chr1: 206,945,163	Intron 1	C>T	36	(6.3)	30	(4.9)	0.298	1.30 (0.79–2.14)
rs3024490	chr1: 206,945,311	Intron 1	T>G	191	(33.2)	224	(36.5)	0.229	0.86 (0.68–1.10)
rs1800872	chr1: 206,946,407	5′ UTR	A>C	180	(31.3)	212	(34.4)	0.245	0.87 (0.68–1.10)
rs1800871	chr1: 206,946,634	5′ UTR	T>C	179	(31.2)	210	(34.1)	0.286	0.88 (0.69–1.12)
rs1800896	chr1: 206,946,897	5′ UTR	A>G	31	(5.4)	40	(6.5)	0.410	0.82 (0.50–1.32)

1: major allele; 2: minor allele.

### Statistical analysis

Hardy–Weinberg equilibrium was tested for each SNP among the controls. Differences in allele and genotype frequencies between cases and controls were assessed by the χ^2^ test. The Haploview 4.1 program was used to compute pair-wise linkage disequilibrium (LD) statistics [24]. Standardized disequilibrium D’ value and r^2^ value were plotted, and LD blocks were defined according to the criteria [25]. Haplotype frequencies were estimated with an accelerated expectation-maximization algorithm, similar to the partition-ligation-expectation-maximization method described previously [26]. P values <0.05 were considered statistically significant.

## Results

We genotyped nine common SNPs in *IL10*: rs1878672, rs1554286, rs1518111, rs3021094, rs3790622, rs3024490, rs1800872, rs1800871, and rs1800896. All SNPs were in Hardy–Weinberg equilibrium in the controls (data not shown). All 9 SNPs were located in 1 haplotype block, and the magnitude of LD between each SNP was extremely high, with pair-wise D’≥0.83 (Figure 1). The allele and genotype frequencies of the 9 SNPs in both the cases and controls are listed in Table 1 and Table2, respectively. No statistically significant association was observed for any of the SNPs between the cases and controls. Furthermore, there were no significant differences in the haplotype frequencies of all 9 SNPs between the cases and controls (Table 3). We analyzed clinical features according to 9 SNPs. In a stratified analysis according to lesion location, which included the eye, lungs, skin heart, and nerves, none of these clinical features were found to be significantly associated with 9 SNPs (data not shown).

**Table 2:** Genotype frequencies of SNPs of the *IL10* gene among cases (n=288) and controls (n=310).

** **	** **	** **	**Genotype Frequency, n (%)**						** **	**Allele 1 Dominant Model, n (%)**				** **	**Allele 1 Recessive Model, n (%)**				** **
**rsID**	**Allele 1>2**	**Status**	**1/1**		**1/2**		**2/2**		**p**	**1/1+1/2**		**2/2**		**p**	**1/1**		**1/2+2/2**		**p**
rs1878672	C>G	Cases	258	(89.6)	29	(10.1)	1	(0.3)	0.650	287	(99.7)	1	(0.3)	0.602	258	(89.6)	30	(10.4)	0.392
		Controls	269	(87.3)	37	(12.0)	2	(0.7)		306	(99.4)	2	(0.7)		269	(87.3)	39	(12.7)	
rs1554286	T>C	Cases	135	(46.9)	125	(43.4)	28	(9.7)	0.640	260	(90.3)	28	(9.7)	0.524	135	(46.9)	153	(53.1)	0.389
		Controls	134	(43.4)	140	(45.3)	35	(11.3)		274	(88.7)	35	(11.3)		134	(43.4)	175	(56.6)	
rs1518111	A>G	Cases	136	(47.2)	124	(43.1)	28	(9.7)	0.574	260	(90.3)	28	(9.7)	0.515	136	(47.2)	152	(52.8)	0.322
		Controls	133	(43.2)	140	(45.5)	35	(11.4)		273	(88.6)	35	(11.4)		133	(43.2)	175	(56.8)	
rs3021094	A>C	Cases	105	(36.5)	142	(49.3)	41	(14.2)	0.0523	247	(85.8)	41	(14.2)	0.236	105	(36.5)	183	(63.5)	0.118
		Controls	132	(42.7)	122	(39.5)	55	(17.8)		254	(82.2)	55	(17.8)		132	(42.7)	177	(57.3)	
rs3790622	C>T	Cases	254	(88.2)	32	(11.1)	2	(0.7)	0.576	286	(99.3)	2	(0.7)	0.524	254	(88.2)	34	(11.8)	0.343
		Controls	279	(90.6)	28	(9.1)	1	(0.3)		307	(99.7)	1	(0.3)		279	(90.6)	29	(9.4)	
rs3024490	T>G	Cases	129	(44.8)	127	(44.1)	32	(11.1)	0.486	256	(88.9)	32	(11.1)	0.343	129	(44.8)	159	(55.2)	0.315
		Controls	125	(40.7)	140	(45.6)	42	(13.7)		265	(86.3)	42	(13.7)		125	(40.7)	182	(59.3)	
rs1800872	A>C	Cases	136	(47.2)	124	(43.1)	28	(9.7)	0.507	260	(90.3)	28	(9.7)	0.438	136	(47.2)	152	(52.8)	0.284
		Controls	132	(42.9)	140	(45.5)	36	(11.7)		272	(88.3)	36	(11.7)		132	(42.9)	176	(57.1)	
rs1800871	T>C	Cases	136	(47.4)	123	(42.9)	28	(9.8)	0.557	259	(90.2)	28	(9.8)	0.524	136	(47.4)	151	(52.6)	0.303
		Controls	133	(43.2)	140	(45.5)	35	(11.4)		273	(88.6)	35	(11.4)		133	(43.2)	175	(56.8)	
rs1800896	A>G	Cases	258	(89.6)	29	(10.1)	1	(0.3)	0.701	287	(99.7)	1	(0.3)	0.601	258	(89.6)	30	(10.4)	0.452
		Controls	269	(87.6)	36	(11.7)	2	(0.7)		305	(99.3)	2	(0.7)		269	(87.6)	38	(12.4)	

1: major allele; 2: minor allele.

**Table 3:** Haplotype frequencies of SNPs of the *IL10* gene among cases and controls.

** **	**Haplotype Frequency, %**		** **	** **
**Haplotype**	**Cases n=288**	**Controls n=310**	**p**	**OR (95%CI)**
**(**rs1878672**, **rs1554286**, **rs1518111**, **rs3021094**, **rs3790622**, **rs3024490**, **rs1800872**, **rs1800871**, and **rs1800896**)**				
CTACCTATA	32.6	32.4	0.934	1.01 (0.79–1.29)
CTAACTATA	28.0	26.1	0.461	1.10 (0.85–1.42)
CCGACGCCA	26.0	27.3	0.605	0.93 (0.72–1.21)
GCGACGCCG	5.4	6.5	0.410	0.82 (0.50–1.32)
CTACTTATA	6.3	4.9	0.304	1.30 (0.79–2.14)
CTAACGATA	1.7	2.5	0.396	0.71 (0.31–1.58)

## Discussion

The aim of the current study was to investigate whether *IL10* polymorphisms affect the development of Japanese patients with sarcoidosis. Our results showed that all the *IL10* polymorphisms were not significantly associated with any clinical subtype of sarcoidosis including ocular involvement in Japanese patients. Here we report a lack of association between *IL10* variants and Japanese sarcoidosis patients, suggesting that the possibility of attributing the pathogenesis of sarcoidosis to *IL10* genetic variations is low.

IL-10 poroduced by Th2 cells suppresses inflammatory cytokines produced by Th1 cells. Although the mechanism of IL-10 in sarcoidosis is unclear, it is thought to be associated with granuloma resolution [2,3]. Some studies have reported that serum levels of IL-10 were increased in several inflammatory diseases, such as; Crohn’s disease, diffuse cutaneous systemic sclerosis and active Behçet's disease [27-29]. In addition, increased serum levels of IL-10 in sarcoidosis patients have been reported [30,31].

Recent studies have also reported that *IL10* gene polymorphisms were associated with several inflammatory diseases. Wang et al. [20] reported IL-10 concentration was significantly higher in Crohn’s disease patients than in the controls and *IL10* polymorphisms were associated with increased patient serum IL-10 levels. Hudson et al. [21] reported that *IL10* genotypes were associated with systemic sclerosis-related autoantibodies and contribute to the etiology of systemic sclerosis. Recently, Mizuki et al. [22] performed a genome-wide association study for Behect’s disease and identified *IL10* as a disease susceptibility gene. Muraközy et al. [32] investigated an association of *IL10* polymorphisms with sarcoidosis, however they could not find any significant differences. As with the previous report, we could not find any association between *IL10* gene polymorphisms and sarcoidosis. On the other hand, Vasakova et al. [33] have recently shown that there are significant differences in the frequencies of *IL10* polymorphisms between sarcoidosis and healthy controls in the Czech Caucasian population, whereas they suggested that their findings cannot be generalized since the sample size in the study was small.

In summary, the *IL10* polymorphisms do not appear to be significantly relevant to Japanese sarcoidosis patients. However, further genetic studies in other ethnic populations are required to elucidate the association between *IL10* polymorphisms and sarcoidosis.
